# Evaluation of the Anti-Inflammatory and Antinociceptive Effects of the Essential Oil from Leaves of *Xylopia laevigata* in Experimental Models

**DOI:** 10.1155/2014/816450

**Published:** 2014-07-03

**Authors:** João Carlos C. Queiroz, Ângelo R. Antoniolli, Lucindo J. Quintans-Júnior, Renan G. Brito, Rosana S. S. Barreto, Emmanoel V. Costa, Thanany B. da Silva, Ana Paula Nascimento Prata, Waldecy de Lucca, Jackson R. G. S. Almeida, Julianeli T. Lima, Jullyana S. S. Quintans

**Affiliations:** ^1^Departamento de Fisiologia, Universidade Federal de Sergipe, 49.000-100 São Cristóvão, SE, Brazil; ^2^Departamento de Química, Universidade Federal de Sergipe, 49.000-100 São Cristóvão, SE, Brazil; ^3^Departamento de Biologia, Universidade Federal de Sergipe, 49.000-100 São Cristóvão, SE, Brazil; ^4^Departamento de Morfologia, Universidade Federal de Sergipe, CEP 49.000-100 São Cristóvão, SE, Brazil; ^5^Colegiado de Ciências Farmacêuticas, Universidade Federal do Vale do São Francisco, 56.304-205 Petrolina, PE, Brazil; ^6^Laboratório de Farmacologia Pré-Clinica (LAPEC), Departamento de Fisiologia, Universidade Federal de Sergipe, Avenida Tancredo Neves, S/N, Bairro, Rosa Elza, 49.000-100 São Cristóvão, SE, Brazil

## Abstract

*Xylopia laevigata* (Annonaceae) is a medicinal plant used in folk medicine to treat pain and inflammation. Thus, we investigated the possible antioxidant, antinociceptive, and anti-inflammatory effects of *X. laevigata* leaf essential oil (EOX) in animal models. Our EOX sample showed the presence of *γ*-muurolene (17.78%), *δ*-cadinene (12.23%), bicyclogermacrene (7.77%), and *α*-copaene (7.17%) as main compounds. EOX presented a strong antioxidant potential according to the DPPH, TBARS, and nitrite production tests. Additionally, pretreatment with EOX, in mice, also significantly produced (*P* < 0.05 or *P* < 0.001) antinociceptive effect by reduction of nociceptive behavior (in formalin and writhing tests). The EOX showed c-Fos label in the olfactory bulb, piriform cortex, and periaqueductal gray. Acute administration of EOX exhibited a significant (*P* < 0.01 or *P* < 0.001) anti-inflammatory profile in the carrageenan-induced peritonitis and by the carrageenan-induced hindpaw edema tests in mice. Our results provide evidence for the use of *X. laevigata* by traditional medicine practitioners in the management of pain and inflammatory disorders.

## 1. Introduction

The Annonaceae is a large family of tropical and subtropical trees and shrubs, comprising about 135 genera and more than 2500 species [1, 2]. This family is known for its edible fruits and the medicinal properties of many of its species [3, 4]. Previous chemical and pharmacological investigations on some species of this family have indicated the presence of important bioactive compounds exhibiting various pharmacological activities, including antidiarrhoeal, analgesic, antimicrobial, insecticidal, antiparasitic, and antitumor properties [5–8].

In the Brazilian Northeast,* Xylopia laevigata* (Mart.) R.E. Fries (Annonaceae) is commonly called “meiú” or “pindaíba,” and it is a plant (leaves and flowers) used popularly to treat painful disorders, heart disease, and inflammatory conditions (oral communications received from local woodsmen known as “mateiros”; data no published). Recently, our group demonstrated anticancer activity of* X. laevigata* essential oil [9]. However, there is little information about* X. laevigata's* biological properties. To the best of our knowledge, this is the first scientific report on the pharmacological properties of the* X. laevigata* species using the antioxidant, analgesic, and anti-inflammatory approaches.

As part of our continuous interest in the Brazilian Northeast's native plants and to support the use of this plant in folk medicine, we investigated the antioxidant, antinociceptive, and anti-inflammatory profiles of the essential oil obtained from the leaves of* X. laevigata *(EOX) through experimental protocols. Additionally, we investigate the effects of EOX on the central nervous system (CNS) areas by immunofluorescence technique to c-Fos.

## 2. Materials and Methods

### 2.1. Chemicals

Acetic acid, Tween 80, formalin, trichloroacetic acid, indomethacin, glycine, luminol (5-amino-2,3-dihydro-1,4-phthalazinedione), TBARS (thiobarbituric acid reactive species), phosphate buffer, sodium nitroprusside, and Griess reagent were purchased from Sigma (USA). Morphine and naloxone were purchased from Cristália (Brazil).

### 2.2. Plant Material

The leaves of* X. laevigata* were collected in March 2010 in the “Serra de Itabaiana,” between the cities of Itabaiana and Areia Branca, Sergipe State, Brazil, at the coordinates [S 10°44′53′′ W 037°20′21′′]. The species were identified by Dr. Ana Paula do Nascimento Prata, plant taxonomist from the Department of Biology (DBI) of the Federal University of Sergipe (UFS). A voucher specimen, number ASE-15440, was deposited at the Herbarium ASE of the UFS (ASE/UFS). The leaves were obtained from flowering species.

### 2.3. Hydrodistillation of the Essential Oil

The essential oil from dried leaves (for 24 h) of* X. laevigata* (200 g) was obtained by hydrodistillation for 3 h using a Clevenger-type apparatus. The essential oil (EOX) was dried over anhydrous sodium sulphate, and the percentage content was calculated on the basis of the dry weight of plant material. The essential oils were stored in a freezer until analysis. The hydrodistillation of the essential oil was performed in triplicate.

### 2.4. GC Analysis

GC analyses were carried out using a Shimadzu GC-17A fitted with a flame ionization detector (FID) and an electronic integrator. Separation of the compounds was achieved employing a ZB-5MS fused capillary column (30 m × 0.25 mm × 0.25 *μ*m film thickness) coated with 5%-phenyl-arylene-95%-methylpolysiloxane. Helium was the carrier gas at 1.2 mL*·*min^−1^ flow rate. The column temperature program was 50°C/2 min, followed by a rate of 4°C/min to 200°C, then a rate of 15°C/min to 300°C, and then a rate of 300°C/15 min [9]. The injector and detector temperatures were 250°C and 280°C, respectively. Samples (0.5 *μ*L in CH_2_Cl_2_) were injected with a 1 : 100 split ratio. Retention indices were generated with a standard solution of* n*-alkanes (C9–C18). Peak areas and retention times were measured by an electronic integrator. The relative amounts of individual compounds were computed from GC peak areas without FID response factor correction.

### 2.5. GC/MS Analysis

GC/MS analyses were performed on a Shimadzu QP5050A GC/MS system equipped with an AOC-20i autoinjector. A J&W Scientific DB-5MS (coated with 5%-phenyl-95%-methylpolysiloxane) fused capillary column (30 m × 0.25 mm × 0.25 *μ*m film thickness) was used as the stationary phase. MS were taken at 70 eV with scan interval of 0.5 s and fragments from 40–500 Da. The other conditions were similar to the GC analysis [12].

### 2.6. Identification of Constituents

EOX components were identified by comparing the retention times of the GC peaks with standard compounds run under identical conditions, by comparison of retention indices [10] and MS with those in the literature [11] and by comparison of MS with those stored in the NIST and Wiley libraries.

### 2.7. Antioxidant Tests

#### 2.7.1. Free Radical Scavenging Activity

The free radical scavenging activity of the extract was determined based on its ability to scavenge the stable DPPH free radical. The experimental protocol was done according to the method described by Leite et al. [13]. Stock solution (10 mg/mL) of the EOX was prepared in EtOH, and serial dilutions were carried out to obtain concentrations of 1, 5, 10, 15, 20, 25, 30, and 35 *μ*g/mL. Diluted solutions (2 mL) were added to 2 mL of a 0.004% EtOH solution of DPPH, mixed, and allowed to stand for 30 min for the reaction to occur. The equation of the concentration × absorbance calibration curve for the DPPH radical was *C* = 110.547 − 0.02804*A*, where *C* is the concentration of the DPPH radical in medium and *A* is the absorbance at 515 nm. The correlation coefficient was* R *= 0.9983. The percentage of remaining DPPH (%DPPHREM) was calculated according to Brand-Williams et al. [14] as follows: %DPPHREM = [DPPH]*T*/[DPPH]*T*0 × 100, where *T* is the time when absorbance was determined (1–60 min) and* T*0 is the zero time. The amount of antioxidant necessary to decrease the initial concentration of DPPH radical by 50% (IC50) was calculated by plotting the percentage of DPPHREM at time of 60 min against various concentrations of EOX. The results were expressed as *μ*g antioxidant/mL DPPH ± standard deviation. Butylated-hydroxytoluene (BHT) was used as the positive control.

#### 2.7.2. TBARS Assay

TBARS assay was employed to quantify lipid peroxidation [15], and an adapted TBARS method was used to measure the antioxidant capacity of EOX using egg yolk homogenate as lipid-rich substrate [16]. Briefly, egg yolk was homogenized (1% w/v) in 20 mM phosphate buffer (pH 7.4), and after that, 1 mL of the homogenate was sonicated and then homogenized with 0.1 mL of EOX at different concentrations. Lipid peroxidation was induced by addition of 0.1 mL AAPH solution (0.12 M). The vehicle used to dilute the EOX (DMSO 10%) was used as the control group. Reactions were carried out for 30 min at 37°C. After cooling, samples (0.5 mL) were centrifuged with 0.5 mL of trichloroacetic acid (15%) at 1200 ×g for 10 min. An aliquot of 0.5 mL from supernatant was mixed with 0.5 mL TBA (0.67%) and heated at 95°C for 30 min. After cooling, sample absorbance was measured using a spectrophotometer at 532 nm. The results were expressed as percentage of TBARS formed by AAPH alone (induced control).

#### 2.7.3. Scavenging Activity of Nitric Oxide (NO)

Nitric oxide was generated from spontaneous decomposition of sodium nitroprusside in 20 mM phosphate buffer (pH 7.4). Once generated, NO interacts with oxygen to produce nitrite ions, which were measured by the Griess reaction [17]. The reaction mixture (1 mL) containing 10 mM sodium nitroprusside (SNP) in phosphate buffer and EOX, at different concentrations, was incubated at 37°C for 1 h. A 0.5 mL aliquot was taken and homogenized with 0.5 mL Griess reagent. The absorbance of chromophore was measured at 540 nm. Percent inhibition of the nitric oxide generated was measured by comparing the absorbance values of negative controls (only 10 mM sodium nitroprusside and vehicle) and assay preparations. Results were expressed as percentage of nitrite formed by SNP alone [16].

### 2.8. Analgesic and Anti-Inflammatory Protocols

#### 2.8.1. Animals

Male Swiss albino mice weighing 28–34 g were housed at 22 ± 2°C under a 12 h light/12 h dark cycle (lights on at 6:00 a.m.), with access to food and water* ad libitum*. The animals were acclimatized for 12 h before testing, and they were used only once throughout the experiments. The experiments were performed according to the Animal Care and Use Committee at the Federal University of Sergipe (CEPA/UFS N° 16/11), and we followed the current guidelines for the care of laboratory animals and the ethical guidelines for investigations of experimental pain in conscious animals [18]. The number of animals (*n* = 6–8, per group) and intensities of noxious stimuli used were the minimum necessary to demonstrate the consistent effects of the drug treatments.

#### 2.8.2. Acetic Acid-Induced Nociception

The abdominal constrictions were induced according to the procedures described previously [19]. The animals were pretreated with EOX at the doses of 12.5, 25, or 50 mg/kg (i.p.) 1 h before injection with acetic acid (0.1 mL/10 g). The control animals (*n* = 8, per group) received a similar volume of vehicle (saline + 2 drops of Tween 80 0.2%), and the standard group was treated with morphine (MOR, 3 mg/kg, i.p.). After the algogen administration, pairs of mice were placed in separate boxes, and the number of abdominal constrictions was cumulatively counted over a period of 15 min. Antinociceptive activity was expressed as the reduction in the number of constrictions in mice pretreated with EOX [16]. Possible antagonism by the EOX or MOR antinociceptive effect was evaluated by pretreatment with naloxone (NAL, 1.5 mg/kg, i.p.), a nonselective opioid antagonist.

#### 2.8.3. Formalin-Induced Pain

We used the Hunskaar and Hole [20] procedure with slight modifications. Nociception was induced by injecting 0.02 mL of 1% formalin in distilled water into the subplantar of the right hindpaw. Mice (*n* = 8, per group) were given EOX (12.5, 25, or 50 mg/kg, i.p.), morphine (MOR, 3 mg/kg), or vehicle 1 h prior to injecting formalin. These mice were individually placed in an acrylic box (25 cm × 15 cm × 15 cm) with mirrors placed on three sides and under the box to enable unhindered observation of the formalin injected paw for assessing the pain reaction time. The time spent paw licking was counted from 0 to 5 min (first phase) and from 15 to 30 min (second phase). These phases represented neurogenic and inflammatory pain responses, respectively [21].

#### 2.8.4. Rotarod Test

To investigate if the treatments with EOX influenced the motor activity of the animals and consequently impaired the assessment of the nociceptive behavior in the experimental models, we evaluated the motor activity of the animals [22]. The mice able to remain on the rotarod apparatus (AVS, Brazil) longer than 180 s (7 rpm) were selected 24 h before the test. Then, the selected animals were divided into five groups (*n* = 6, per group) and treated i.p. with vehicle (control), EOX (12.5, 25, or 50 mg/kg), or diazepam (DZP, 3 mg/kg). One hour later, each animal was tested on the rotarod apparatus, and the time (s) they remained on the bar for up to 180 s was recorded after 60 min.

#### 2.8.5. Leukocyte Migration to the Peritoneal Cavity Test

Leukocyte migration was induced by injection of carrageenan (500 *μ*g/cavity, i.p., 500 *μ*L) into the peritoneal cavity of mice (*n* = 6, per group) 1 h after administration of EOX (12.5, 25, or 50 mg/kg, i.p.) or indomethacin (INDO, 10 mg/kg, i.p.) by modification of the technique previously described by [23]. The mice were euthanized by cervical dislocation 4 h after carrageenan injection. Shortly afterwards, phosphate buffered saline (PBS) containing EDTA (1 mM, i.p., 10 mL) was injected. Immediately, a brief massage was done for further fluid collection, which was centrifuged (2000 rpm, 5 min) at room temperature. The supernatant was disposed and 1 mL of PBS was introduced to the precipitate. An aliquot of 10 *μ*L from this suspension was dissolved in 200 *μ*L Turk solution, and the total cells were counted in a Neubauer chamber, under optical microscopy. The results were expressed as the number of leukocytes/mL. The percentage of leukocyte inhibition = (1 − *T*/*C*) × 100, where* T* represents the treated group leukocyte count and* C* represents the control group leukocyte count.

#### 2.8.6. Carrageenan-Induced Mice Paw Edema

This test was used to determine the anti-inflammatory action of EOX by the method described by [24], with alterations. Groups of 8 mice received, by the intraperitoneal route, either 10 mg/kg INDO (reference drug), EOX (12.5, 25 or 50 mg/kg), or vehicle (saline + 2 drops of Tween 80 0.2%). One hour before this, they had been given a subplantar injection of 0.1 mL/paw of carrageenan solution (200 mg/kg) suspended in distilled water into the right hindpaw. The mouse paw volume up to the ankle joint was measured using a plethysmometer (LE 7500, PanLab, Spain) at 0 (just before) and 3 h after the injection of carrageenan. The increase in paw edema volume was considered as the difference between 0 and 3 h.

#### 2.8.7. Immunofluorescence

Ninety minutes after the EOX intraperitoneal injections at doses 12.5, 25, and 50 mg/Kg the animals (*n* = 4 per group) were anesthetized (ketamine 100 mg/Kg and xylazine 10 mg/Kg) and submitted to transcardial perfusion with phosphate buffer (0.01 M) saline isotonic (PBS) followed by formalin 10% buffered 0.1 M (pH 7.4) and brains were collected, cryoprotected, and stored at −80°C with Tissue-Tek O.C.T. (Sakura-USA) for immunofluorescence processing to c-Fos.

Frozen serial transverse sections of 20 *μ*m containing the olfactory bulb, piriform cortex, and periaqueductal gray were collected on gelatinized glass slides. The tissue sections were stored at –80°C until use. The sections were washed with PBS 3 times for 5 minutes and incubated with 0.1 M glycine in PBS for 10 minutes. Nonspecific protein binding was blocked by incubation of the sections for 30 minutes in a solution containing 1% bovine serum albumin (BSA). After that, the sections were incubated overnight with rabbit polyclonal c-Fos antibody (sc52 from Santa Cruz Biotech., USA) as primary antibodies. Afterwards, the sections were incubated for one hour with donkey polyclonal antibody to rabbit IgG conjugated with Alexa Fluor 594 as secondary antibodies (Life Technologies, USA). The cover slip was mounted with Fluoromount G (Electron Microscopy Sciences, USA). As an immunofluorescence control for nonspecific labeling (negative control; (data no shown)), brain sections were incubated without primary antibody. As positive control (data no shown), it was processed for immunofluorescence brain sections containing the supraoptic nucleus from an animal previously submitted to osmotic stimulus (2 mL/100 g of animal weight) with hypertonic saline (0.5 M) instead of EOX. After each stage, slides were washed with PBS 3 times for 5 minutes.

### 2.9. Acquisition and Analyses of Images

An average number of the c-Fos positive cells of the fifteen pictures from a piece studied nucleus (olfactory bulb, piriform cortex, and periaqueductal gray) acquired (Axioskop 2 plus, Carl Zeiss, Germany) for each animal were calculated. Four animals were used to each tested dose of EOX. The olfactory bulb, piriform cortex, and periaqueductal gray were classified in agreement with the description of Paxinos and Watson Atlas (1997). Neurons were counted of the whole studied nucleus by Image J (National Institute of Health) using a plugin (written by authors described at http://www.lb.ufs.br/lcb/index.php/turorial-for-imagej) that use the same level of label intensity to select and count the c-Fos positive cells.

### 2.10. Statistical Analysis

The data obtained were evaluated by analysis of variance (ANOVA), either one- or two-way, followed by Tukey's or Fisher's tests. In all cases, differences were considered significant if *P* < 0.05. All statistical analyses were done using GraphPad Prism 3.02 (GraphPad Prism Software Inc., SanDiego, CA, USA). Percent inhibition of edema volume between treated and control group was calculated using the following formula: Inhibition % = 100 · (Vc − Vt)/Vc, where Vc and Vt represent mean increase in paw volume in control and treated groups, respectively.

## 3. Results 

### 3.1. Chemical Composition

Hydrodistillation of the leaves of* X. laevigata* gave a light-yellowish crude essential oil (EOX), with a yield of 1.58% (w/w), in relation to the dry weight of the plant material. As shown in Table 1, it was possible to identify 36 compounds. The EOX was constituted predominantly by sesquiterpene compounds at 91.30%. The major compounds identified were *γ*-muurolene (17.78%), *δ*-cadinene (12.23%), bicyclogermacrene (7.77%), *α*-copaene (7.17%), germacrene D (6.54%), (*E*)*-*caryophyllene (5.87%), *γ*-cadinene (4.72%), aromadendrene (4.66%), and *γ*-amorphene (4.39%).

In addition to the major constituents, limonene (3.36%), *α*-cubebene (3.04%), germacrene B (2.86%), spathulenol (2.29%), *β*-copaene (1.86%), *α*-ylangene (1.26%), *α*-pinene (1.25%), *β*-cubebene (1.20%), and *α*-muurolol (1.11%) have been reported in the essential oils of several other species of* Xylopia* (Maia et al., 2005), indicating that this species is a typical member of the Annonaceae family.

The amount of DPPH radical that reacted with EOX (30 *μ*g/mL, 30 min) was 98.15% (Table 2). The essential oil presented a response similar to the positive control, butylated-hydroxytoluene (BHT) (30 *μ*g/mL, 30 min).

**Figure 1:**
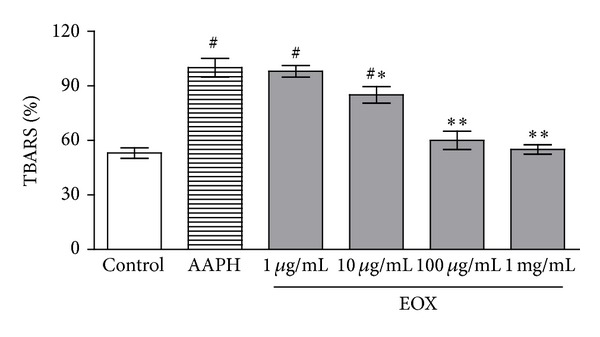
TBARS* in vitro*. Lipid extracted from egg yolk was subjected to oxidative damage by incubation with AAPH, and the ability of different concentrations of EOX to prevent TBARS formation was analyzed. Control means basal lipid peroxidation with vehicle alone (DMSO 10%); AAPH alone group is considered as 100% of oxidative damage. Values represent mean ± S.E.M., *n* = 4, experiments in triplicate. ^#^ *P* < 0.001versus control; _ _*P < 0.05 and _ _**P < 0.001 different versus AAPH group (system) (ANOVA followed by Tukey's test).

To assess the antioxidant potential of EOX, we tested its ability to prevent oxidative damage to lipids induced by a free radical source* in vitro* (AAPH). Quantification by TBARS demonstrated that EOX exerts a significant (*P* < 0.05 or *P* < 0.001) antioxidant effect against peroxyl radicals generated by AAPH, protecting lipids from oxidation in a dose-dependent fashion (Figure 1).

**Figure 2:**
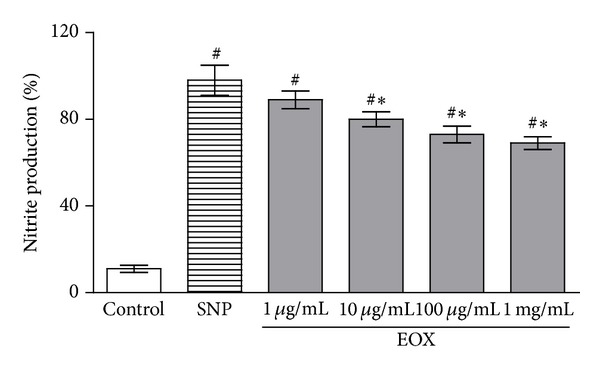
NO-scavenging activity. Control means basal NO production of vehicle (DMSO 10%) in the absence of a NO generator source (without SNP); SNP group reflects nitrite production by sodium nitroprusside alone, considered 100% of NO production. The effect of different concentrations of EOX against SNP was determined by the Griess method. Values represent mean ± S.E.M., *n* = 4, experiments in triplicate. ^#^ *P* < 0.001 versus control; _ _*P < 0.01 different versus SNP (ANOVA followed by Tukey's test).

To determine the ability of EOX to act as a reactive nitrogen species (RNS) scavenger, we evaluated the NO-scavenging activity by incubating EOX with sodium nitroprusside (SNP), a chemical inducer of NO production. EOX at 10 *μ*g/mL, 100 *μ*g/mL, and 1 mg/mL showed a significant NO-scavenging activity with *P* < 0.01 (Figure 2).

As shown in Table 3, EOX at the doses of 12.5, 25, or 50 mg/kg (i.p.) significantly inhibited (*P* < 0.01 or *P* < 0.001) the acetic acid-induced writhings and two phases of formalin-induced nociception in mice. Pretreatment with naloxone did not reverse the effect of EOX (50 mg/kg, i.p.) but antagonized the antinociceptive effect of MOR on the acid-induced writhing response.

In the rotarod test, EOX treated mice did not show any significant motor performance alterations with the doses of 12.5, 25, or 50 mg/kg (data no shown). As might be expected, diazepam (3 mg/kg, i.p., standard drug) significantly reduced the time of treated animals on the rotarod apparatus when compared with the control group.

Carrageenan (500 *μ*g/cavity) induced leukocyte migration to the peritoneal cavity 4 h after stimulus.  Table 4 shows the inhibitory effect of EOX on carrageenan-induced responses in a dose-dependent manner (33.5, 36.4, and 42.4% at doses of 12.5, 25, and 50 mg/kg, resp.; *P* < 0.01 or *P* < 0.001). Additionally, the mean increase in paw edema volume was about 48.3 ± 2.8 mL in the vehicle-treated control mice. EOX (25 and 50 mg/kg, i.p.) significantly (*P* < 0.01) reduced the mean paw edema volume at 3 h after carrageenan injection. The standard drug, indomethacin (INDO, 10 mg/kg, i.p.), showed highly significant (*P* < 0.001) anti-inflammatory activity in both inflammatory tests (Table 4).

**Figure 3:**
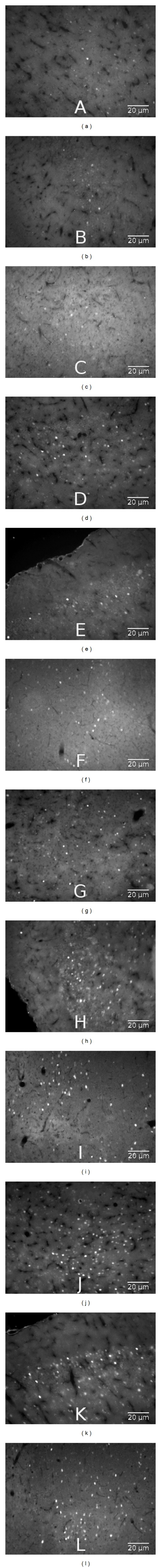
Immunofluorescence staining of nuclear c-Fos in the neurons of the olfactory bulb (a, d, g, and j), piriform cortex (b, e, h, and k), and periaqueductal gray (c, f, i, and l) 90 minutes after* X. laevigata* leaf essential oil (EOX) intraperitoneal injection at doses of 0 (saline control), 12.5, 25, and 50 mg/Kg, respectively, on these three brain regions of the animals submitted to carrageenan hypernociception model.

In the olfactory bulb and in the piriform cortex (Figure 3 and Table 5) of the animals, the average number of neurons showing c-Fos was increased by an intraperitoneal injection of EOX at doses of 25 and 50 mg/Kg when compared with control (vehicle). However, the intraperitoneal injections of EOX at dose of 12.5 mg/Kg did not change the average number of neurons showing c-Fos when compared with control (vehicle).

In the periaqueductal gray (Figure 3 and Table 5) of the animals, the average number of neurons showing c-Fos was increased by an intraperitoneal injection of EOX at doses of 12.5, 25, and 50 mg/Kg when compared with control (vehicle).

## 4. Discussion

There is little information about the biological properties of* Xylopia laevigata*. In fact, we did a search using Chemical Abstracts, Biological Abstracts, Web of Science, and ScienceDirect (updated to December 2013) and did not find any article on the same pharmacological properties of this species investigated in our work. We believe that it is the first scientific report on the antioxidant, analgesic, and anti-inflammatory profiles of the* X. laevigata* species and which sought to map the CNS areas involved in analgesic profile. So, the aim of the study was to evaluate the antioxidant, antinociceptive, and anti-inflammatory potential of the essential oil from the leaves of* X. laevigata *(EOX) in experimental protocols.

Antioxidants comprise a broad and heterogeneous family of compounds that share the common task of interfering with (stopping, retarding, or preventing) the oxidation (or autoxidation) of an oxidizable substrate [25]. Numerous physiological and biochemical processes in the human body may produce oxygen-centered free radicals and other reactive oxygen or nitrogen species as by-products [26]. Overproduction of such radicals can cause oxidative damage to biomolecules, eventually leading to many diseases, such as atherosclerosis, cancer, diabetes, or inflammatory conditions and pain [16, 27]. Recently, there has been growing interest in research into the role of plant-derived antioxidants in food and human health [28].

Initially, the capacity for scavenging free radicals was evaluated for EOX through the DPPH test. This test is a very convenient method for screening small antioxidant molecules because the intensity of reaction can be analyzed by simple spectrophotometric assay [13]. DPPH radical is scavenged by antioxidants through the donation of hydrogen to form the stable, reduced DPPH molecule. Thus, the antioxidant radicals are stabilized through the formation of nonradical products [29]. EOX produced a potent antioxidant effect.

Nitric oxide (NO) exerts important physiological effects, such as vasoconstriction regulation and modulation of proinflammatory processes [30]. Reaction between some ROS (notably superoxide) and NO is generally fast and may result in “cleaning” of NO in some cells, with consequent inhibition of some NO-triggered biological effects [16, 30]. So, NO plays an important role in various types of inflammatory disorders and thus might be implicated in the antinociceptive and anti-inflammatory actions shaped by EOX.

Lipid peroxidation has been described as the biological damage caused by free radicals that are formed under oxidative stress [31]. Several plants have been shown to inhibit lipid peroxidation, as measured by TBARS results [28, 32, 33]. The lipids in membrane are continuously subjected to oxidant challenges. Oxidant-induced abstraction of a hydrogen atom from an unsaturated fatty-acid chain of membrane lipids initiates the process of lipid peroxidation, which propagates as a chain reaction [32]. In the process, cyclic peroxides, lipid peroxides, and cyclic end peroxides are generated, which ultimately are fragmented into aldehydes such as malondialdehyde [26]. This antioxidant profile can be useful for managing disorders, as inflammation and pain [16, 32, 34].

In evaluation of the antinociceptive effect using acetic acid-induced abdominal constriction, EOX produced a significant inhibition of the nociceptive behavior. This test is a standard, simple, and sensitive test for measuring analgesia induced by both opioids and peripherally acting analgesics [35]. In the present study, our results suggest that EOX has a central analgesic effect. To confirm such an effect, we tested the blocking effect of naloxone, a specific antagonist of morphinomimetic receptors, in the acetic acid test [36]. Naloxone was not able to completely reverse the antinociceptive effect of EOX but appears to partially reverse this effect, suggesting a weak involvement of opioid receptors. According to Le Bars et al. [21], in the acetic acid test, pain is elicited by the injection of an irritant, such as acetic acid into the peritoneal cavity, which produces episodes of characteristic stretching (writhing) movements; those behavioral changes are probably in relation to the inhibition in the peritoneal fluid levels of prostaglandin and cytokines [21, 37]. Thus, EOX may also participate in the inhibition of prostaglandin synthesis, as nociceptive mechanisms involve the processing or release of arachidonic acid metabolites via COX and prostaglandin biosynthesis [16].

Acute administration of EOX, at all doses, caused pronounced antinociception as evidenced by the decreased nociceptive behavior in the first and second phases of the formalin test. This test model is sensitive to various classes of analgesic drugs [20] and is characterized by the first phase (neurogenic), which is evoked by direct formalin stimulation of the sensorial C-fibers followed by substance P release [38], and the second phase (inflammatory) mainly due to a subsequent inflammatory reaction in the peripheral tissue mediated by the release of various inflammatory mediators associated with the increased level of PG, induction of COX, and release of nitric oxide (NO) [21]. As EOX reduced the production of nitrite, showing it to have a potential role as a NO scavenging agent, and as NO plays an important role in various types of inflammatory processes, it is thus possible that the reduction of NO is involved in a potential antinociceptive action produced by EOX, mainly in the second phase of the formalin test [39].

To investigate if treatments with EOX could influence the motor activity of the animals and consequently impair the assessment of the nociceptive behavior in the experimental models, the motor activity of the animals was evaluated on a rotarod apparatus [16]. Our results revealed that all mice treated with EOX, at the doses evaluated, did not show any performance alteration during the rotarod test (data no shown).

To demonstrate central action of EOX, c-Fos labeled by immunofluorescence was performed showing activation of olfactory bulb, piriform cortex, and periaqueductal gray (PAG). As demonstrated in immunohistochemistry results, the acute treatment with EOX stimulated the olfactory bulb, piriform cortex, and PAG. The olfactory bulb and piriform cortex receive information from such brain areas as the amygdala and hippocampus and project their axons to targets in the amygdala and hypothalamus, where they may influence aggressive, mating, and painful behavior [40, 41]. Following the report of Reynolds [42], the PAG was rapidly established as being important to descend inhibition of spinal nociceptive processing and also as a site where opioids, when microinjected directly into the PAG, replicated the inhibitory effects of electrical stimulation [43]. It also became clear that stimulation in widespread sites in the brain, including the sensory cortex, thalamus, hypothalamus, midbrain, pons, and spinal cord, similarly produced inhibitory effects on spinal nociceptive processing. Additionally, stimulation of PAG area can produce descending inhibitory of pain effects [44]. Thus, our results allow us to suggest that the EOX may be acting by modulation of the descending pain-inhibitory mechanisms. Actually, recent studies have suggested that essential oils and/or terpenes can produce effects on descending pain pathways, as PAG and nucleus raphe magnus areas [45, 46].

To evaluate its anti-inflammatory profile we seek to test EOX in different models. Carrageenan has been widely used as a noxious agent able to induce experimental inflammation for the screening of compounds possessing anti-inflammatory activity [47]. This phlogistic agent, when injected locally into a rodent paw, produces a severe inflammatory reaction, which is discernible within 30 min [48]. As described by Bhandare et al. [47], the development of edema induced by carrageenan is a biphasic event; the early phase of the inflammation is due to the release of histamine, serotonin, and similar substances; the later phase is associated with the activation of kinin-like substances and the release of prostaglandins, proteases, and lysosome [49]. EOX inhibited hindpaw edema and showed considerable anti-inflammatory activity. In addition, the antioxidant action of EOX observed in the TBARS and NO assays suggests that this essential oil may act as a protective agent against oxidative damage to membrane polyunsaturated fatty acids (PUFAs), such as arachidonic acid, which is a very important component in the response to inflammation via the cyclooxygenase (COX) pathway [30].

Thus, to confirm the probable anti-inflammatory profile of EOX, the peritonitis induced by carrageenan was observed. Cell recruitment during inflammation depends on the orchestrated release of local mediators that are responsible for local vascular and tissue changes as well as for the recruitment of host defense cells [50]. The inflammation induced by carrageenan involves cell migration, plasma exudation, and production of mediators, such as nitric oxide, prostaglandin E2, interleukin (IL)-1*β*, IL-6, and tumor necrosis factor (TNF)-*α* [44]. These mediators are able to recruit leukocytes, such as neutrophils, in several experimental models. EOX inhibited leukocyte migration induced by i.p. injection of carrageenan (in the peritonitis model) in a dose-dependent manner. In addition, it is possible that EOX, rich in hydrophobic molecules, interacts strongly with specific types of lipids, and in a lipid-rich system, such as in the TBARS assay, lipids with lesser affinity to EOX compounds and/or hydrophilic portions of amphipathic lipids are more susceptible to radical attack, allowing the initiation of a lipoperoxidation chain reaction [17], and these effects may be contributing to the anti-inflammatory property of EOX.

Additionally, Martins et al. [51] suggest that the antioxidant and anti-inflammatory effects of the essential oil of* Garcinia brasiliensis *may be related to the presence of *γ*-muurolene and *δ*-cadinene, two terpenoids rich in our oil. Unfortunately, we cannot isolate these terpenoids in EOX to test them separately. Even so, it is possible that the pharmacological properties, such as anti-inflammatory and analgesic, of EOX are related to the presence of terpenoid compounds [52–54].

## 5. Conclusions

For the first time in literature, our results support that the EOX exhibits an antioxidant action preventing lipoperoxidation, NO release, and significant anti-inflammatory and antinociceptive activities in rodents. EOX probably exerts its antinociceptive effect by central inhibitory mechanisms (with partial involvement of the opioid system) and does not produce changes in motor coordination.

The probable anti-inflammatory activity of EOX may play a role in interfering with prostaglandin synthesis and also might involve redox-mediated mechanisms. Also, this study demonstrated, for the first time, that EOX has relevant central antinociceptive property probably due to the involvement of descending modulation of pain on CNS, as PAG. The current findings support its medicinal use in the folk medicine of the Brazilian Northeast as an analgesic and anti-inflammatory medicine.

## Figures and Tables

**Table 1:** Essential oil composition from the leaves of *X. laevigata*.

Compound		RI^a^	RI^b^	*X. laevigata* leaf oil %
1	*α*-Pinene	931	932	1.25
2	*β*-Pinene	975	974	0.40
3	Limonene	1027	1024	3.36
4	(Z)-*β*-Ocimene	1034	1032	0.38
5	*δ*-Elemene	1333	1335	0.65
6	*α*-Cubebene	1345	1345	3.04
7	*α*-Ylangene	1367	1373	1.26
8	*α*-Copaene	1374	1374	7.17
9	*β*-Bourbonene	1381	1387	0.51
10	*β*-Cubebene	1386	1387	1.20
11	(*E*)-Caryophyllene	1417	1417	5.87
12	*β*-Copaene	1427	1430	1.86
13	Aromadendrene	1436	1439	4.66
14	*Trans*-Muurola-3,5-diene	1447	1451	0.33
15	*α*-Humulene	1453	1452	0.83
16	Alloaromadendrene	1458	1458	0.25
17	*Cis*-Cadina-1(6),4-diene	1460	1461	0.17
18	*Trans*-Cadina-1(6),4-diene	1469	1475	Tr
19	*γ*-Muurolene	1474	1478	17.78
20	Germacrene D	1480	1484	6.54
21	*δ*-Selinene	1485	1492	0.17
22	*γ*-Amorphene	1490	1495	4.39
23	Bicyclogermacrene	1494	1500	7.77
24	*α*-Muurolene	1496	1500	Tr
25	*δ*-Amorphene	1503	1511	0.13
26	*γ*-Cadinene	1511	1513	4.72
27	*δ*-Cadinene	1516	1522	12.23
28	*Trans*-Cadina-1,4-diene	1530	1533	0.33
29	*α*-Cadinene	1534	1537	1.09
30	*α*-Calacorene	1539	1544	0.63
31	Germacrene B	1557	1559	2.86
32	*β*-Calacorene	1560	1564	Tr
33	Spathulenol	1575	1577	2.29
34	Caryophyllene oxide	1580	1582	0.65
35	*α*-Muurolol	1642	1644	1.11
36	*δ*-Cadinol	1653	1649	0.81
	Monoterpenes			5.39
	Sesquiterpenes			91.30
	Total identified			**96.69**

RI^a^ (calc.), retention indices on DB-5MS column calculated according to van Den Dool and Dec. Kratz (1963) [10]. RI^b^ retention indices according to Adams (2007) [11]. Tr: trace.

**Table 2:** Radical scavenging activity of *X. laevigata* leaf essential oil (EOX) determined by the reduction of DPPH free radical.

Samples	IP (%)	IC_50_ (*μ*g/mL DPPH)
EOX	98.15	11.98 ± 0.55∗
BHT	99.26	11.06 ± 0.61∗

*n* = 4. ∗IC_50_ and IP (30 *μ*g/mL) of extracts were calculated at the steady state (30 min).

**Table 3:** Effect of *X. laevigata* leaf essential oil (EOX) or morphine (MOR), in the absence and presence of naloxone (NAL), on writhing induced by acetic acid and formalin-induced nociception tests in mice.

Treatment	Dose (mg/kg)	Writhing test	Formalin test	
		Number of writhings^a^	0–5 min^a^	15–30 min^a^
Vehicle	—	38.0 ± 4.1	79.5 ± 5.6	105.1 ± 23.3
EOX	12.5	24.8 ± 6.2	48.2 ± 5.7^b^	41.5 ± 12.9^c^
EOX	25	8.7 ± 3.4^c^	36.3 ± 6.7^c^	31.7 ± 8.3^c^
EOX	50	3.1 ± 2.8^c^	30.3 ± 7.2^c^	13.8 ± 6.6^c,e^
MOR	5	1.9 ± 0.6^c^	12.4 ± 2.8^c^	5.8 ± 1.1^c^
EOX + NAL	50 + 1.5	21.5 ± 6.1^b,d^	—	—
MOR + NAL	5 + 1.5	34.8 ± 7.3	—	—

*n* = 8, per group. ^a^Values represent mean S.E.M. ^b^ *P* < 0.05 (one-way ANOVA and Tukey's test), significantly different from control group. ^c^ *P* < 0.001 (one-way ANOVA and Tukey's test), significantly different from control group. ^d^ *P* < 0.01 (one-way ANOVA and Tukey's test), significantly different from EOX 50 mg/kg group. ^e^ *P* < 0.05 (one-way ANOVA and Tukey's test), significantly different from EOX 25 mg/kg group.

**Table 4:** Effect of *X. laevigata* leaf essential oil (EOX) or indomethacin (INDO) on carrageenan-induced leukocyte migration and carrageenan-induced hindpaw edema in mice.

Treatment	Dose (mg/kg)	Carrageenan-induced leukocyte migration (leukocytes ×10^6^/mL)^a^	% inhibition	Carrageenan-induced hindpaw edema volume (mL)^a^	% inhibition
Vehicle	—	48.3 ± 2.8	—	0.61 ± 0.14	—
EOX	12.5	32.1 ± 3.6^b^	33.5^d^	0.40 ± 0.11^b^	34.4^d^
EOX	25	30.7 ± 2.2^b^	36.4^d^	0.36 ± 0.09^b^	41.0^d^
EOX	50	27.8 ± 4.3^c^	42.4^d^	0.34 ± 0.11^b^	44.3^d^
INDO	10	19.7 ± 2.5^c^	59.2^e^	0.21 ± 0.08^c^	65.6^e^

*n* = 6, per group. ^a^Values represent mean ± S.E.M. ^b^ *P* < 0.01 (one-way ANOVA and Tukey's test), significantly different from control. ^c^ *P* < 0.001 (one-way ANOVA and Tukey's test), significantly different from control. ^d^ *P* < 0.01 (Fisher's test), significantly different from control. ^e^ *P* < 0.001 (Fisher's test), significantly different from control.

**Table 5:** Brain areas activated by *X. laevigata* leaf essential oil (EOX) in mice.

Treatment	Dose	FOS positive cells		
	(mg/kg)	Olfactory bulb	Piriform cortex	Periaqueductal gray
Vehicle	—	3.5 ± 0.6	3.0 ± 1.4	4.2 ± 1.3
EOX	12.5	11.7 ± 2.6	8.0 ± 1.7	23.7 ± 5.2^a^
EOX	25	22.0 ± 5.4^a^	29.5 ± 3.6^b^	28.7 ± 1.1^b^
EOX	50	25.7 ± 6.4^b^	34.0 ± 9.4^b^	19.2 ± 3.6^a^

*n* = 4, per group; values represent mean ± S.E.M. ^a^ *P* < 0.01 or ^b^ *P* < 0.001 (one-way ANOVA and Tukey's test), significantly different from vehicle-treated mice.
